# Dysfunctions in the Mature Dendritic Cells Are Associated with the Presence of Metastases of Colorectal Cancer in the Surrounding Lymph Nodes

**DOI:** 10.1155/2016/2405437

**Published:** 2015-12-29

**Authors:** Anna Pryczynicz, Dariusz Cepowicz, Konrad Zaręba, Mariusz Gryko, Joanna Hołody-Zaręba, Bogusław Kędra, Andrzej Kemona, Katarzyna Guzińska-Ustymowicz

**Affiliations:** ^1^Department of General Pathomorphology, Medical University of Bialystok, Waszyngtona 13, 15-276 Białystok, Poland; ^2^2nd Department of General and Gastroenterological Surgery, Medical University of Bialystok, M. Sklodowskiej-Curie 24 A, 15-276 Białystok, Poland; ^3^Department of Gynecology and Obstetrics, Śniadecki Memorial Hospital, M. Sklodowskiej-Curie 26, 15-276 Białystok, Poland

## Abstract

Dendritic cells play a key role in the antigen presentation and T cell activation. The aim of this study was a detailed analysis of the presence of mature dendritic cells (CD 83 positive) in colorectal cancer in correlation with selected clinicopathological parameters. The presence of mature dendritic cells (mDCs) was determined immunohistochemically using the anti-CD83 antibody. The morphometric analysis of the mDCs was performed in the normal colon wall adjacent to the cancerous tumor as well as in the front of the tumor and in the main mass of the cancerous tumor. Decrease in mDCs in the front and in the main tumor mass was observed. The increase in the number of mDCs in both of these locations was associated with the presence of metastases in the nearby lymph nodes (*p* < 0.05 and *p* < 0.01). Furthermore, the increase in the proportion of mDCs in the main tumor mass was associated with the presence of the invasion of tumor cells into the blood and lymph vessels (*p* < 0.01). The increase in the amount of mDCs in the cancerous tumor is associated with the invasiveness of the tumor and especially with the metastasis to the surrounding lymph nodes.

## 1. Introduction

Dendritic cells (DCs) are a group of highly specialized cells which play an important role in the initiation and modulation of the immune response. They are a heterogeneous group and they are divided into two subpopulations on the basis of differences in the expression of surface antigens. The first is the subtype of myeloid dendritic cells, responsible for the cell mediated immunity and the stimulation of the differentiation of T lymphocytes towards the Th1 helper lymphocytes. They are equipped with a network of protrusions through which they interact with other surrounding cells and also the extracellular matrix. The second subpopulation is the subtype of plasmacytoid dendritic cells, which are responsible for innate as well as acquired immunity [1–4]. They are commonly found in almost all organs and structures, where they dwell in the immature stage. In this form they are capable of recognizing and absorbing foreign antigens through endocytosis, macropinocytosis, or phagocytosis [1]. The maturation process of dendritic cells consists of the change of their function towards antigen presentation to T lymphocytes. Inter alia, endogenous cytokines, substances of bacterial origin, remnants of dying cells, or heat shock proteins are responsible for activating the maturation of DC [5, 6].

The participation of dendritic cells in various human diseases including malignant tumors is very broadly described. The mechanism of the escape of tumor cells from immunological control is based, inter alia, on the inhibition of the maturation and functioning of the dendritic cells. Cancer cells have the capacity to release factors such as IL-10, VEGF, or TGF-*β*, which inhibit the maturation of DC [7, 8]. Therefore, in tumors of the circulatory system of the nipple, lung tumors, and other tumors, a decrease in the number of dendritic cells is observed [9–11].

The aim of this study was a detailed analysis of the presence of mature dendritic cells (CD83 positive) in colorectal cancer in correlation with selected clinicopathological parameters.

## 2. Methodology

### 2.1. The Studied Group

The study group consisted of 71 patients treated surgically due to colon cancer at the Department of Gastroenterological Surgery at the Medical University of Białystok. Patients did not receive preoperative chemotherapy or radiotherapy. The material was fixed in 10% buffered formalin and secured in formalin. In a routine histological evaluation of clippings stained with hematoxylin and eosin, the histological type and the anatomo-clinical stage (pTNM) were analyzed. The features of pT, pN, and pM were determined on the basis of a histopathological picture in correlation with clinical data. The presence of distant metastases has been established on the basis of appropriate radiological examination and fine-needle aspiration. Furthermore, the presence of buds according to Guzińska-Ustymowicz [12], the presence of inflammatory infiltration within the tumor, and the presence of tumor cell emboli in blood vessels and lymphatic vessels according to Jass were assessed [13].

### 2.2. Immunohistochemical Analysis

Formalin-fixed and paraffin-embedded tissue specimens were cut on a microtome into 4 *μ*m sections. The sections were deparaffinized in xylenes and hydrated in alcohols. To visualize the antigen, the sections were heated in a microwave oven for 20 min in a citrate buffer (pH = 6.0) in the temperature of 97°C. They were incubated with 3% hydrogen peroxide solution in order to block endogenous peroxidase. Next, incubation was performed with mouse monoclonal antibody against human CD83 (CD83 clone 1H4b, Novocastra NCL-CD83, Poland) for 1 hour at room temperature. The reaction was carried out using biotinylated anti-mouse antibody and streptavidin-conjugated with horseradish peroxidase (LSAB2, DAKO, Poland). A color reaction for peroxidase was developed with chromogene DAB (DAKO, Poland). The detailed morphometric analysis of the mDCs was performed in the normal colon wall adjacent to the cancerous tumor as well as in the front of the tumor and in the main mass of the cancerous tumor according to Suzuki et al. [14]. CD83 positive cells were counted by two independent pathomorphologists in 3 representative fields of view at a magnification of 400x.

### 2.3. Statistical Analysis

The statistical analysis of the comparison of two groups was performed on the basis of the *U* Mann-Whitney test. The relationships between the number of CD83 positive cells and clinicopathological factors were determined using Spearman's correlation coefficient test. A *p* value of <0.05 was considered statistically significant.

## 3. Results

### 3.1. Histological Localization of Mature Dendritic Cells (CD83+) in the Normal Mucous Membrane and in the Colorectal Cancer

**Figure 1:**
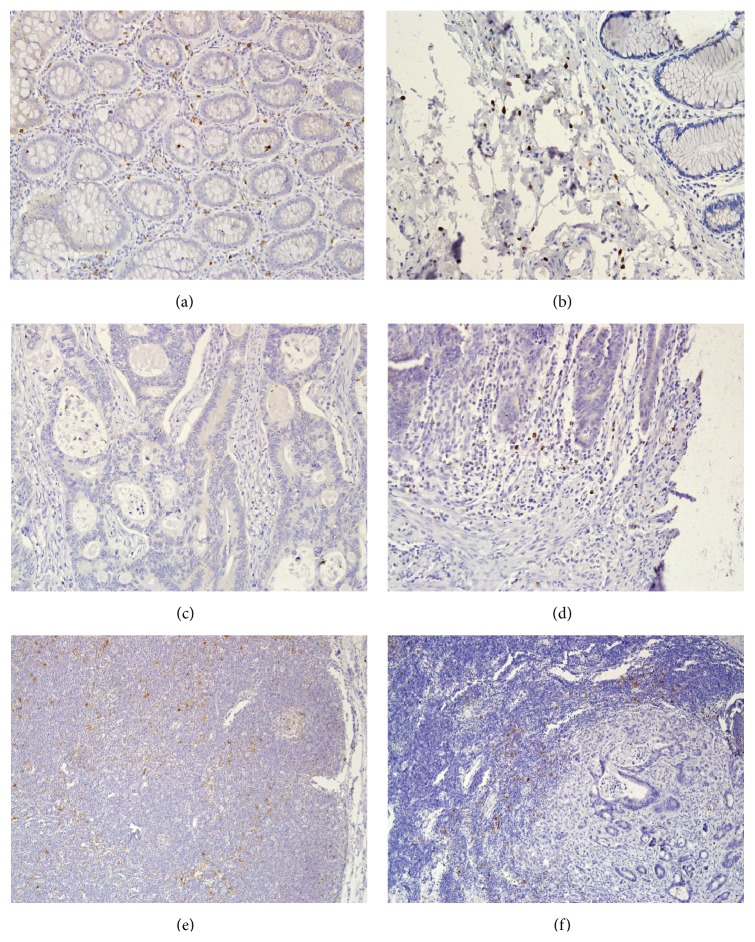
Mature dendritic cells immunohistochemically stained with the CD83 antibody present in (a) the stroma of the normal colorectal mucous membrane (magnification ×200); (b) the submucosal membrane of the large intestine (magnification ×200); (c) the stroma of the main mass of the tumor (magnification ×200); (d) the front of the tumor (magnification ×200); (e) the normal lymph node (magnification ×100); and (f) the lymph node affected with metastases (magnification ×100).

In the stroma of the normal colon mucous membrane, up to 40 mature dendritic cells were observed in the visual field (an average of approximately 13 cells). Similarly, in the submucosal membrane about 10 mDCs were observed, fewer in the muscle layer, about 5 in the serosa about 2 and in the fatty tissue about 3 mature dendritic cells in the visual field. In the stroma of the main mass of the tumor the absence or very few mature dendritic cells (0–8 cells, an average of up to one cell in the visual field) were observed. However, in the front of the tumor an increase of CD83 positive cell numbers of even up to 17 in the field of view (on average around 5 mature dendritic cells) was observed (Figure 1). The differences in the quantities of mature dendritic cells in normal mucous membrane in the front of the tumor and in the main mass of the tumor were statistically significant (Table 1).

### 3.2. Analysis of the Amount of CD83+ Cells in the Front and the Main Mass of the Tumor in Correlation with Selected Clinic Pathological Parameters

There was no significant dependency observed between the number of mature dendritic cells in the front of the tumor and in the main mass of the tumor and the age and sex of the patient, the location of the tumor, the histological type, cancer pT stage, the presence of distant metastases, budding, and inflammatory infiltration. It was noted, however, that the increase in the number of mature dendritic cells in the front of the tumor and in the main mass of the tumor is associated with the presence of metastasis in the surrounding lymph nodes (*p* < 0.05 and *p* < 0.01, resp.) (Table 2). Furthermore, the increase in the proportion of the mature dendritic cells in the main mass of the tumor was associated with the presence of the invasion of the tumor cells into the blood and lymph vessels (*p* < 0.01).

## 4. Discussion

Our research indicates a significant decrease in the number of mature dendritic cells in the stroma of colorectal cancer. In the normal colorectal mucous membrane adjacent to the cancerous tumor we observed quite numerous CD83 positive cells. Their much smaller number, but still larger than in the main mass of the tumor, was recorded in the front of the tumor. These observations confirm previous reports in the literature [14–16]. Yuan et al. [15] evaluated the concentration of mature dendritic cells also in colorectal adenomas. The number of mDCs in them was smaller than in the normal tissue and comparable to the number of mDCs in the front of the colorectal cancer, whereas the number of mDCs in the stroma of the main mass of the tumor was significantly lower. It is still not explained what the cause of the decline in the number of mDCs in the colorectal cancer is. One of the theories explaining the nonhomologous distribution of mDCs in the tumor is the assumption that they move to the surrounding lymph nodes in order to present antigen [15]. In our study we observed a significant density of mDCs in reactive lymph nodes located near the tumor. This theory, however, requires a detailed confirmation. Yuan et al. [15] in their studies also observed that in the tumor tissue there is a strong concentration of immature dendritic cells as compared to normal colorectal mucous membrane. However, their maturation may be impaired by the presence in the stroma of the tumor of various inhibiting factors secreted by the tumor cells. The theory of the inhibited maturation and migration of the dendritic cells in the cancerous tumor appears to be more likely and is now the subject of research [17–19].

We also examined the relationship of the density of mDCs in the front of the tumor as well as in the main mass of the tumor of the colorectal cancer with clinic pathological parameters. We have demonstrated that the increase in the number of mDCs in both locations is associated with the presence of metastases in the surrounding lymph nodes. Furthermore, the increase in the number of mDCs in the main mass of the tumor is associated with the presence in the blood vessels and lymphatic vessels of infiltration from cancer cells. This may indicate a stronger response of the dendritic cells as a result of the growth of the aggressiveness of the tumor. We did not, however, find similar correlations reported in the literature. In the reactive lymph nodes collected from the area of the tumor, we observed numerous mDCs evenly distributed in the lymph node (Figure 1(e)), whereas, in the lymph node with cancer metastasis, the mDCs were mainly located on the border of the lymphatic and cancer tissue, although their number was not significantly different from the normal lymph node (Figure 1(f)). McMullen et al. [20] have conducted detailed studies on the density of dendritic cells in the surrounding lymph nodes in rectal cancer. According to their studies, in the lymph nodes of the rectum there are a large number of mDCs, much higher than in the mucous membrane of the rectum. On the other hand, in the lymph nodes adjacent to the cancerous tumor, an exhaustion of the mDCs occurs, but regardless of the stage of the tumor. Furthermore, the number of mDCs in the front of the tumor did not differ depending on the presence of metastases in the surrounding lymph nodes, in contrast to our results. Also Gulubova et al. [21] did not observe differences in the density of CD83 positive cells and the presence of metastases in the regional lymph nodes. They observed, however, a decrease in mDCs in the tumors with a more invasive histological type, locally advanced (T3-T4), and in tumors with the presence of metastases to distant organs. What is more, it was found that patients with a reduced number of CD83 positive cells in the front of the cancerous tumor or in the metastases of the colorectal cancer have worse survival rates in comparison to patients with a higher infiltration of mature dendritic cells [21, 22].

Reassuming, our studies confirm the decrease in the number of mDCs in the colorectal cancer, which is associated with the escape of the tumor cells from the human immune system. Furthermore, the increase in the amount of mDCs in the cancerous tumor is associated with the invasiveness of the tumor, especially with the metastasis to the surrounding lymph nodes. However, this process needs to be clarified in more detailed studies.

## Figures and Tables

**Table 1:** The number of CD83 positive cells in normal tumor tissue, in the front of the tumor, and in the main mass of the tumor in colorectal cancer.

	*N*	Number of CD83 positive cells			
		Mean	Median	SD	Range
Correct mucous membrane^a^	42	13.3	10.5	9.55	0–40
Front of the tumor^b^	71	5.05	4	3.87	0–17
Main tumor mass^c^	71	0.85	0	1.71	0–8

a versus b, *p* < 0.001; a versus c, *p* < 0.001, b versus c, *p* < 0.001.

**Table 2:** The correlations between the number of CD83 positive cells in the front of the tumor and in the main mass of the tumor and the clinicopathological parameters.

			Number of CD83 positive cells			
Parameter		*N*	In invasion front		In main mass of tumor	
			Mean (range)	*p* value (coefficient)	Mean (range)	*p* value (coefficient)
Age	<60	14	5.5 (0–17)	NS	0.2 (0–3)	NS
	≥60	57	4.9 (0–16)		1.0 (0–8)	

Gender	Male	41	4.9 (0–17)	NS	0.8 (0–8)	NS
	Female	30	5.2 (0–16)		0.9 (0–8)	

Localization	Colon	40	5.1 (0–16)	NS	1.0 (0–8)	NS
	Rectum	31	5.0 (0–17)		0.6 (0–6)	

Adenocarcinoma type	Nonmucinous	64	4.9 (0–17)	NS	0.9 (0–8)	NS
	Mucinous	7	5.8 (2–12)		0.1 (0-1)	

pT stage	1	1	3.0 (3)	NS	2.0 (2)	NS
	2	4	4.7 (0–9)		0.7 (0–3)	
	3	64	5.1 (0–17)		0.8 (0–8)	
	4	2	3.5 (3-4)		1.0 (0–2)	

Lymph node metastasis	Absent	42	4.2 (0–16)	**<0.05 (0.278)**	0.5 (0–8)	**<0.01 (0.308)**
	Present	29	6.2 (0–17)		1.2 (0–8)	

Distant metastasis	Absent	59	5.2 (0–17)	NS	0.9 (0–8)	NS
	Present	12	4.4 (0–11)		0.2 (0–2)	

Tumor budding	Absent	18	4.3 (0–16)	NS	0.8 (0–8)	NS
	Present	53	5.3 (0–17)		0.8 (0–8)	

Lymphocytic infiltration	0	11	5.1 (0–15)	NS	1.3 (0–8)	NS
	1	33	5.2 (0–17)		0.6 (0–6)	
	2	20	4.9 (0–16)		1.0 (0–8)	
	3	7	4.5 (2–9)		0.4 (0–2)	

Vascular invasion	Absent	33	4.7 (0–16)	NS	0.6 (0–8)	**<0.01 (0.312)**
	Present	38	5.3 (0–17)		1.0 (0–8)	

The statistically significant results were indicated with bold type. NS: statistically nonsignificant.
